# Deprescribing of proton pump inhibitors in internal medicine in-patients: A retrospective observational study

**DOI:** 10.1016/j.rcsop.2026.100796

**Published:** 2026-05-07

**Authors:** Christina Kotoula, Leonie Daria Schreck, Arani Anandarathakrishnan, Carla Meyer-Massetti, Carole Elodie Aubert, Manuel Haschke, Evangelia Liakoni

**Affiliations:** a Clinical Pharmacology and Toxicology, Department of General Internal Medicine, Inselspital, Bern University Hospital, University of Bern, Bern, Switzerland; b Graduate School for Health Sciences, University of Bern, Switzerland; c Institute of Primary Health Care (BIHAM), University of Bern, Bern, Switzerland; d Department of General Internal Medicine, Inselspital, Bern University Hospital, University of Bern, Bern, Switzerland

**Keywords:** Deprescribing, Proton pump inhibitors, Medication appropriateness, Polypharmacy, Pharmacoepidemiology

## Abstract

**Background:**

Proton pump inhibitors (PPI) treat gastrointestinal disorders and are often overprescribed or continued unnecessarily, despite potential long-term adverse effects (e.g. infections). Deprescribing is recommended when indications are unclear or treatment unnecessarily prolonged. In Switzerland, national guidelines on PPI deprescribing are lacking. This study assessed PPI deprescribing frequency, appropriate new prescriptions and possible reasons for not performing deprescribing.

**Methods:**

Retrospective study at the Inselspital, Bern, Switzerland. Included were patients ≥18 years admitted to internal medicine wards during January–February 2022 with PPI on admission or newly prescribed during hospitalization. PPI duration and indication were retrieved from electronic health records. Data were evaluated with descriptive statistics and multivariable regression models.

**Results:**

Among 852 admissions, 54% (*n* = 458) were included (38% female, 72% ≥60 years old). Of them, 70% (*n* = 320) had PPI on admission and 30% (*n* = 138) newly prescribed during hospitalization, with appropriate use in 48% (153/320) and 49% (68/138) cases, respectively. At discharge, 376 (82%) of the included cases had PPI, 44% (*n* = 166) with appropriate use. Among 300 patients with indicated deprescribing, this occurred in 90 (30%) and was less likely with PPI on admission (OR = 0.2, 95% CI:0.1–0.4, *p* < 0.01). Possible reasons for not performing deprescribing (*n* = 210) included unmet bleeding prophylaxis criteria (61%, *n* = 128), unclear re-evaluation plans (8%, *n* = 17) and outdated indications (7%, *n* = 15).

**Conclusion:**

Most PPI were continued upon discharge, despite half lacking a clearly appropriate use. Knowledge, communication and information gaps may have contributed to missed deprescribing opportunities. Regular medication reviews across healthcare settings could improve deprescribing practices and ensure appropriate PPI use.

## Highlights

• More than half of the cases lacked a clear proton pump inhibitor (PPI) indication
• Nearly all continued the PPI during hospitalization and at discharge
• Deprescribing was less likely in patients already admitted with a PPI

## 1 Introduction

Proton pump inhibitors (PPI) block gastric acid production by inhibiting the H+/K + -ATPase on the luminal side of the parietal cell membrane. They are commonly used to treat peptic ulcer or gastroesophageal reflux disease (GERD) and for *helicobacter pylori* eradication. In general, PPI are well tolerated and thus often prescribed longer than necessary^1^, ^2^, ^3^. In Switzerland, for example, the PPI pantoprazole belongs to the four most frequently purchased active substances.^4^ Adverse effects potentially associated with chronic PPI use, including enteric infections, malabsorption of minerals and vitamins, osteoporosis-related bone fractures, and renal and cardiovascular disease are infrequent; however, due to the widespread use, can lead to a high absolute number of affected patients.^1^, ^5^ Furthermore, a potential drug-drug interaction leading to reduced antiplatelet effect of the pro-drug clopidogrel, which is activated by cytochrome P450 enzymes (CYP), has been discussed.^6^, ^7^ Such an interaction may be clinically important given the high prevalence of antiplatelet therapy, particularly in older patients with cardiovascular comorbidities who frequently receive PPI for gastrointestinal protection, and the potentially life-threatening consequences in case of antiplatelet treatment failure. However, data are inconsistent and the relevance of this interaction remains controversial.^7^, ^8^, ^9^, ^10^ PPI may also reduce the absorption of some medications (e.g. certain tyrosine kinase or HIV protease inhibitors) and the elimination of high-dose methotrexate.^11^, ^12^, ^13^ Thus, deprescribing should be regularly evaluated to avoid potential adverse effects and drug-drug interactions while reducing the financial burden on the healthcare system for unnecessary medication in such a large group of patients.^14^

Deprescribing can be defined as “*the process of withdrawal of an inappropriate medication, supervised by a health care professional with the goal of managing polypharmacy and improving outcomes*”.^15^ Although this evaluation should take place regularly to optimize patient safety, adherence and cost-effectiveness, the process can be time consuming, requires experience with complex pharmacotherapies and can be hindered if more than one physician is involved. Additional challenges include inaccurate or incomplete information in the medical history with no corresponding documented diagnoses for some of the prescribed medications.^16^

In some countries, specific algorithms17., 18 and guidelines are available to support PPI deprescribing.19., 20 However, such coordinated efforts are not yet established in Switzerland and data on the prevalence of and reasons for inadequate PPI prescribing needed to develop effective strategies and implement preventive measures are sparse. The aim of this project was to determine the frequency of PPI deprescribing during hospital stay and on discharge among internal medicine patients at a large hospital in Switzerland, as well as appropriate new prescriptions during hospitalization. We additionally investigated possible reasons for not performing deprescribing, as well as the most frequent appropriate indications.

## 2 Methods

### 2.1 Design and setting

Observational retrospective chart review using electronic health records of patients admitted to the internal medicine wards of the Inselspital, University Hospital, Bern, Switzerland, in January and February 2022, regardless of discharge date. Included were all patients 18 years or older with either already established PPI treatment on admission or PPI newly prescribed during the hospital stay, regardless of the dose used. The study was classified as quality assurance project and thus waived from approval by the local ethics committee of the canton Bern (Req. 2023–00098). Reporting is based on the *Strengthening the Reporting of Observational Studies in Epidemiology* (STROBE) guidelines.

### 2.2 Data extraction

The cases were extracted by the Insel Data Science Center (IDSC). Data included patients' demographics (e.g. age, sex), month and year of hospitalization, comedication, length of hospital stay, outcome (discharge, transfer to another hospital ward, or death), the PPI agent used, route of administration and duration of therapy. The electronic health records of the extracted cases were reviewed for documented indications for PPI use and whether the PPI was discontinued. For patients with PPI therapy on admission, the treatment duration was determined using available medical records from previous admissions within six months prior to the index hospitalization. Where PPI therapy was started during the hospital stay, appropriateness of use was investigated, as well as whether any information regarding intended duration of PPI therapy was provided in the discharge report. For included patients admitted more than once within the study period, each hospitalization was treated as a separate case.

### 2.3 Data cleaning

Indications and duration for PPI therapy were evaluated using a list created based on the official Swiss product information^21^ and recommendations in recent guidelines and expert consensus reports^18^, ^20^, 21., 22., 23, 24, 25, 26, 27, 28., 29, 30 (Table 1).

**Table 1:** Indications and therapy duration considered appropriate for therapy with proton pump inhibitors (PPI)^16^, 19., 20, 21., 22., 23, 24, 25, 26, 27, 28., 29

Indications	Therapy duration
• *Helicobacter pylori* (*H. pylori*) eradication (triple-therapy^a^)/gastritis type B	2 weeks
• Erosive gastroesophageal reflux disease (GERD) grade A or B according to Los Angeles classification • Reflux symptoms, acid regurgitation, heartburn/stomach burn • Non-erosive gastroesophageal reflux disease (NERD) • Duodenal/ gastric ulcer • Ulcer bleeding after endoscopy • NSAID^b^ associated erosion/ gastritis type C • Gastritis (unspecified), erosive antral gastritis, congestive gastritis • Upper abdominal complaints • Dyspepsia (including functional acid-related dyspepsia) • Irritable stomach • Gastropathy • Stress ulcer prophylaxis during intensive care unit stay • Axial hiatus hernia • Esophageal varices	≤8 weeks
• Pathological hypersecretion (idiopathic, Zollinger-Ellison syndrome) • Barrett's esophagus • Eosinophilic esophagitis • Peptic esophageal stricture/ stenosis • Erosive GERD grade C or D according to Los Angeles classification • Prophylaxis of progression of idiopathic pulmonary fibrosis • Prophylaxis after relapse/recurrence after discontinuation with: ➢ GERD ➢ Dyspepsia • Prevention of recurrence of idiopathic duodenal/ gastric ulcer • Previous gastrointestinal ulcer bleeding • Bleeding prophylaxis from the following combinations: ➢ NSAID (incl. aspirin and coxibs^c^) AND at least one of the following: ▪ Age ≥ 60 years ▪ Second NSAID ▪ Anticoagulants^d^ ▪ Corticosteroids^e^ ➢ Antiplatelet drugs^f^ AND at least one of the following: ▪ Aspirin (dual antiplatelet therapy) ▪ Anticoagulant ▪ Age ≥ 60 years ▪ Corticosteroids ▪ GERD symptoms ▪ Dyspepsia ▪ NSAID	>8 weeks

a Triple therapy: PPI+ clarithromycin+ second antibiotic (amoxicillin or metronidazole).

b NSAID (non-steroidal anti-inflammatory drugs): acetylsalicylic acid (aspirin), diclofenac, ibuprofen, flurbiprofen, dexibuprofen, naproxen, indometacin, ketorolac, piroxicam, acemetacin, dexketoprofen, mefenamic acid, nimesulide, tenoxicam, etodolac.

c Coxibs (COX-2-inhibitors): etoricoxib, celecoxib.

d Anticoagulants: direct oral anticoagulants (dabigatran, rivaroxaban, apixaban, edoxaban), vitamin K antagonists (acenocoumarol, phenprocoumon), heparin incl. unfractionated & low molecular weight (dalteparin, enoxaparin, nadroparin, fondaparinux).

e Corticosteroids: betamethasone, budesonide, dexamethasone, hydrocortisone, methylprednisolone, prednisolone, prednisone, triamcinolone.

f Antiplatelet drugs: acetylsalicylic acid (aspirin), clopidogrel, prasugrel, ticagrelor, cangrelor, eptifibatid, tirofiban.

Although some studies define increased bleeding risk at ≥65 years,^18^, ^20^, ^26^ we used the lowest age threshold reported in the literature (≥60 years).^25^, 28. A case was considered “appropriate PPI use” if both documented indication and duration were appropriate. For unclear cases, a dual-reviewer approach was implemented to achieve consensus.

If no appropriate PPI indication or therapy duration were documented, they were considered missing. Other undocumented information (e.g. medication lists, admission or discharge reports) was recorded as missing and appropriate use could not be evaluated. Bleeding prophylaxis was rarely explicitly documented as reason for PPI use, but it was considered appropriate indication if the criteria defined in Table 1 were met on admission or at discharge based on the available information. Co-medications were reviewed manually on admission and at discharge. Polypharmacy was defined as the use of ≥5 medications,^31^ excluding medications taken only when required (“pro re nata”). When different formulations of the same active substance were prescribed, these were counted as a single medication. Fixed-dose combination products were also counted as one medication. In the absence of records prior to hospitalization, the duration was considered appropriate for long-term PPI indications (>8 weeks), while for short-term indications (≤8 weeks), the duration was considered missing information. Deprescribing was either assessed as “yes” (PPI stopped or tapered during hospital stay, plan to stop/ re-evaluate PPI with a specified date documented), “no” (neither performed nor planned), “no need” (for adequate long term PPI indications), while patients who died were assessed as “deceased”. Eligible patients for deprescribing included those for whom PPI were deprescribed (“yes”) and those for whom deprescribing was needed but not performed or planned (“no”).

### 2.4 Statistical analysis

Non-normally distributed continuous data are presented as median and range, nominal data as number of cases and proportion (%). The frequency of appropriate newly prescribed PPI during hospitalization was calculated as the proportion of cases with appropriate PPI use among those with newly PPI prescriptions. PPI deprescribing frequency was calculated by dividing cases where the PPI was deprescribed by the number of cases eligible for deprescribing (deceased patients and cases categorized as “no need to stop/taper PPI” were excluded from this calculation). Potential factors associated with PPI deprescribing were investigated with a binary logistic regression model, with the deprescribing status (yes vs. no) for patients eligible for deprescribing as outcome and age, sex, PPI on admission, duration of hospitalization and polypharmacy on admission as independent variables.^32^, ^33^ Age was categorized into ≥60 (cut-off used for prophylactic PPI treatment) vs. 18–59 years for the regression model.^25^, 28. The duration of hospitalization in days was included as continuous variable and the linearity with the logit of the outcome was investigated by Box-Tidwell approach. The model was exploratory, with multi-collinearity assessed using the variance inflation factors (VIF <4 indicating no multi-collinearity). A *p*-value <0.05 was considered statistically significant. Analyses were conducted using the GNU R statistical software (version 4.5.0) and data visualization was performed with GraphPad Prism (v.8.0.1) and GNU R.

## 3 Results

During the 2 months of the study period, 852 admissions (830 patients) were recorded to the internal medicine wards and 458 cases (54%; 442 patients) had a PPI on admission and/or during hospitalization. Among the included cases, 175 (38%) were female and 331 (72%) were ≥ 60 years old. Additional case characteristics can be found in Table 2.

**Table 2:** Characteristics of internal medicine patients with proton pump inhibitors (PPI) on admission or newly started during hospitalization (*n* = 458).

	**n (%) or median (range)**
**Sex**	
Male	283 (62)
Female	175 (38)
**Age (years)**	72 (18–96)
18–39	36 (8)
40–59	91 (20)
60–74	127 (28)
≥75	204 (44)
**Hospitalization duration (days)**	6 (1−102)
**Medications on admission**	8 (0−23)
No medications/ no list available	55 (12)
1–4	64 (14)
5–9	173 (38)
10+	166 (36)
**Medications at discharge**	9 (0–34)
No medications	25 (5)
1–4	48 (10)
5–9	167 (36)
10+	218 (48)
**Outcome**	
Discharged	308 (67)
Transferred to a different ward/hospital	128 (28)
Deceased	22 (5)

**Fig. 1:**
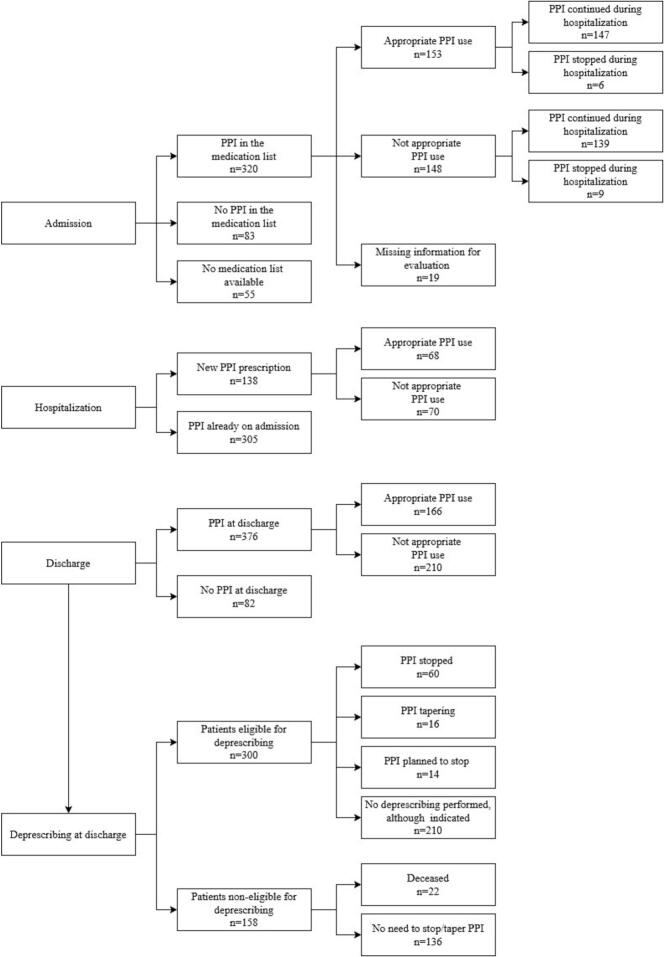
Flow chart of included cases (PPI: proton pumps inhibitors).

**Fig. 2:**
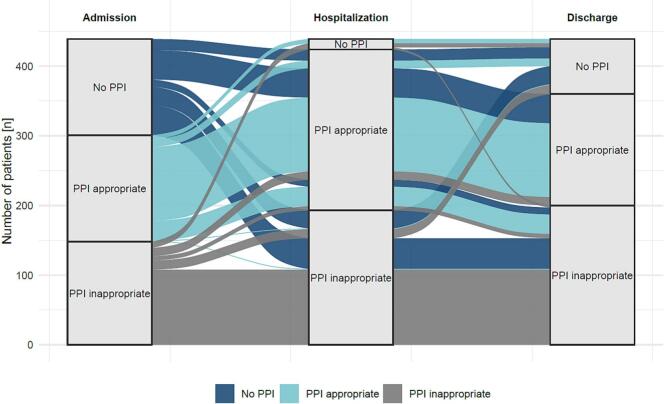
Alluvial plot showing the patient trajectories of proton pump inhibitors (PPI) use and appropriateness on admission, during hospitalization and at discharge. Flow width is proportional to the number of patients in each category. The number of patients with different trajectories are provided as supplementary material.  (PPI-appropriate: appropriate PPI indication & duration, PPI-inappropriate: not appropriate PPI indication and/or duration).

Among the 458 admissions, 320 (70%,) had a PPI on their medication list on admission, with 48% (*n* = 153) among them having appropriate use. Among the 148 patients without appropriate use (46%), 119 (80%) did not have an appropriate indication and 29 (20%) had an inappropriate therapy duration. During hospitalization, 443 cases (97%) received a PPI. Of these, 31% (*n* = 138) were newly PPI prescriptions, 56% (*n* = 248) had an appropriate use and of the 196 (44%) inappropriate use cases, 87% (*n* = 170) lacked an appropriate indication and 13% (*n* = 26) an appropriate duration. At discharge, 376 of the total 458 admissions (82%) had a PPI, 44% (*n* = 166) with appropriate use. Overall, 56% (*n* = 210) of the 376 cases were classified as inappropriate due to missing appropriate indication (*n* = 146, 70%) or treatment duration (*n* = 64, 30%). Among the 138 new PPI prescriptions during hospitalization, 49% (*n* = 68) were classified as appropriate based on the available documentation. Of the 300 patients eligible for deprescribing at discharge, deprescribing was performed in 90 (30%). Further details on PPI use and deprescribing are provided in Table 3 and as flowchart in Fig. 1; Fig. 2 presents an overview of the patient trajectories regarding PPI use.

**Table 3:** Overview of proton pump inhibitors (PPI) use and deprescribing on admission, during hospitalization and at discharge.

	**n (%) or median (range)**
**Admission**	
**PPI on medication list** (n = 458)	
Yes	320 (70)
No	83 (18)
No medication list	55 (12)
**Duration of PPI treatment** (n = 320)	
No information	84 (26)
<2 weeks	16 (5)
<8 weeks	36 (11)
>8 weeks	46 (14)
>6 months	138 (43)
**Appropriate PPI use** according to available documentation (n = 320)	
Yes	153 (48)
No	148 (46)
Not enough information	19 (6)
**Hospitalization**	
**PPI during hospitalization** (n = 458)	
Yes	443 (97)
PPI already on admission	305 (69)
PPI new prescription	138 (31)
PPI deprescribed	15 (3)
**PPI duration** in days (*n* = 443)	6 (1–102)
**PPI appropriate use** according to available documentation (*n* = 443)	
Yes	248 (56)
No	195 (44)
**PPI agent (n = 443)**	
Pantoprazole	342 (77)
Esomeprazole	80 (18)
Omeprazole	1 (<1)
Esomeprazole/ pantoprazole (switch)	17 (4)
Dexlansoprazole /pantoprazole (switch)	1 (<1)
Esomeprazole/ omeprazole (switch)	1 (<1)
Omeprazole/ pantroprazole (switch)	1 (<1)
**PPI route of administration (n = 443)**	
Oral	387 (87)
Intravenous	12 (3)
Oral & intravenous	44 (10)
**Discharge**	
**PPI on medication list (**n = 458)	
Yes	376 (82)
No	82 (18)
**Information on duration after discharge** (*n* = 376)	
<2 weeks	11 (3)
<8 weeks	12 (3)
>8 weeks	4 (1)
Tapering	17 (4)
No information	332 (88)
**PPI appropriate use** according to available documentation (n = 376)	
Yes	166 (44)
No	210 (56)
**Deprescribing at discharge** (n = 458)	
PPI stopped	60 (13)
PPI tapering	16 (4)
PPI planned to stop	14 (3)
No need to stop/taper PPI	136 (30)
No deprescribing performed, although indicated	210 (46)
Deceased	22 (5)

**Fig. 3:**
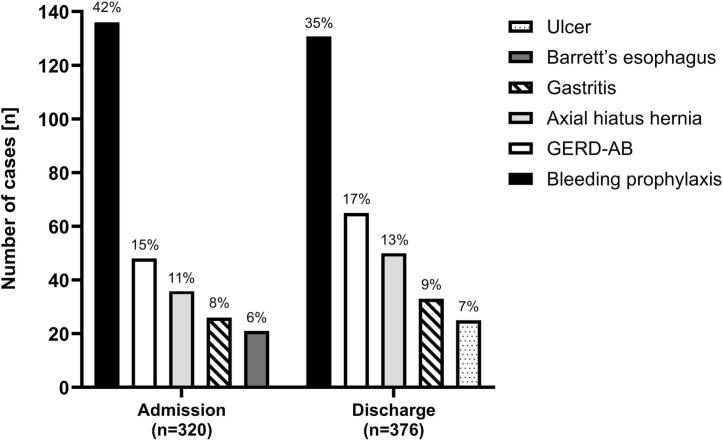
Most frequent appropriate proton pumps inhibitor (PPI) indications on admission and at discharge. (Gastritis: type C, unspecified, erosive antral and congestive gastritis; GERD-AB: Erosive gastroesophageal reflux disease grade A or B according to Los Angeles classification; Ulcer: duodenal/gastric ulcer and ulcer bleeding after endoscopy).

The most frequent PPI indication was bleeding prophylaxis (*n* = 136 on admission and *n* = 131 at discharge) and mild symptoms of GERD during hospitalization (*n* = 67). Fig. 3 presents the most frequent appropriate indications on admission and at discharge; in the supplementary material all cases with appropriate indications are listed for each phase.

In total, 128 of 210 cases (61%), in which deprescribing was indicated but not performed, did not fulfill the bleeding prophylaxis criteria and no further indication for PPI use was documented. Among the 192 cases discharged with an anticoagulant and a PPI, 38% (*n* = 72) received a PPI without meeting the criteria for bleeding prophylaxis or having any other documented indication. Similarly, of the 98 cases with corticosteroid prescriptions at discharge, 45% (*n* = 44) did not have an appropriate PPI prescription based on the available documentation. Conversely, in 12 of 131 (9%) cases that met bleeding prophylaxis criteria, a PPI was not prescribed despite being indicated. Possible misinterpretations of indications based on comments in the discharge reports and observed practices contributing to a lack of deprescribing are summarized in Table 4.

**Table 4:** Potential misinterpretations and practices based on the medical records leading to deprescribing not being performed (n = 210).

	**n (%)**
No documented PPI therapy duration	199 (95)
Bleeding prophylaxis criteria not met (e.g. only corticosteroids (n = 44), anticoagulants (n = 72), NSAID (*n* = 10), aspirin (n = 2)) and no further indication reported	128 (61)
Medication list changed from admission to discharge, bleeding prophylaxis criteria not met anymore at discharge and re-evaluation of PPI not performed	21 (10)
*Re*-evaluation of PPI indication delegated to post hospital care provider (e.g. general practitioner, after gastroscopy) without specifying the date	17 (8)
Appropriate indication in patient history, but prolonged therapy without re-evaluation	15 (7)
Inappropriate indication (e.g. nausea, vomiting, cancer, chemotherapy, prophylaxis of esophagus fistula formation, upside-down stomach)	10 (5)
Missed information (e.g. gastroscopy findings never shared, PPI re-evaluation never performed after no findings in gastroscopy, transfer between reports missed documentation of therapy duration and indication)	3 (1)
No indication mentioned (appropriate or inappropriate), but continuation of PPI instructed	3 (1)

NSAID: Non-steroidal anti-inflammatory drugs (aspirin is reported separately as it can be prescribed either as NSAID or antiplatelet agent); PPI: proton pumps inhibitor.

The binary regression model showed that deprescribing was less likely to be performed in patients already admitted with a PPI (OR = 0.23, 95% CI:0.12–0.45, *p* < 0.01). Female sex (OR = 0.58, 95%CI: 0.33–1.01, *p* = 0.05), age < 60 years old (OR = 0.70, 95% CI: 0.39–1.25, *p* = 0.23), duration of hospitalization (OR = 0.99, 95% CI: 1.00–1.03, *p* = 0.77) and polypharmacy on admission (OR = 1.35, 95% CI: 0.67–2.71, *p* = 0.40) were not significantly associated with deprescribing.

## 4 Discussion

In this retrospective chart review of internal medicine patients hospitalized in a large tertiary Swiss hospital, only half of the patients with a new PPI prescription during hospitalization had an appropriate use based on the documented indication and duration of treatment. Most patients with a PPI on admission or newly started during hospitalization were also discharged with a PPI and the deprescribing frequency while hospitalized was low. Potential reasons for not performing deprescribing included incomplete adherence to criteria for bleeding prophylaxis (e.g. treatment with an anticoagulant, a corticosteroid or a NSAID alone without an additional risk factor), deferral of PPI re-evaluation to other healthcare professionals (e.g. general practitioner) and failure to verify appropriateness of continued PPI treatment for pre-existing diagnoses. PPI deprescribing was less likely in patients already admitted with a PPI, possibly indicating reluctance to stop an established therapy even if no clear indication is documented. The most common adequate PPI indications were bleeding prophylaxis, axial hiatus hernia, mild GERD symptoms and Barrett esophagus.

PPI are among the main drug classes involved in overuse and inappropriate prescribing not only in Western high-income countries^34^ but also in low- and middle- income countries.^35^ Analyses of insurance claims data in Switzerland showed a significant increase, not only of the annual PPI use,^36^ but also of the potentially inappropriate PPI prescriptions between 2012 and 2017.^37^ In our study, based on the available documentation, almost half of the patients receiving a PPI during hospitalization had an inappropriate use and in the majority of the cases no treatment duration was documented in the discharge report. This aligns with other studies that also found a lack of documented indication for PPI use in approximately half of the hospitalized and primary care patients in Western countries.^38^, ^39^, ^40^, ^41^, ^42^ Similarly, PPI were identified as the most prevalent potentially inappropriate medications in China.^43^

In recent years, substantial efforts have been made to develop evidence-based guidelines for appropriate PPI use and deprescribing. In 2014, The National Institute for Health and Care Excellence (NICE) published guidelines for GERD and dyspepsia, recommending annual PPI review and deprescribing when appropriate.^19^ In 2017, Farrell et al. proposed a comprehensive PPI deprescribing algorithm, covering both short- and long-term therapies^18^ and a few years later, the American Gastroenterological Association published recommendations for deprescribing PPIs in the outpatient setting.^20^ In addition to these specific guidelines, general deprescribing tools such as the START/STOPP tool^44^, ^45^ and the Beers criteria^46^ have been developed, as well as more specific initiatives such as the “Bye-bye, PPI” campaign by the Choosing Wisely Canada.^47^ Since the introduction of these guidelines, deprescribing rates have increased, with one study reporting higher PPI discontinuation in recent years (16% in 2019–2022) compared with earlier periods (4% in 2015–2018).^48^ In our study, PPI was deprescribed in nearly one-third of patients, which is higher than rates reported in a previous study.^48^ A Swiss cross-sectional study published in 2019 evaluating the appropriateness of PPI use according to NICE guidelines found that 13% of hospitalized patients assessed on a single day had undergone deprescribing.^41^ Similarly, a multicenter multinational study reported that deprescribing occurred in 20% of a selected subpopulation of older patients (≥70 years) with at least three chronic conditions and polypharmacy (≥5 medications) recruited in 2016–2018, using international classification of disease (ICD) and anatomical therapeutic chemical (ATC) codes to identify indications.^49^ These findings suggest that increased awareness of deprescribing guidelines in combination with the recognition of potential adverse long-term adverse effects and concerns about healthcare costs may have improved deprescribing practices. Nevertheless, substantial opportunities remain to further reduce inappropriate PPI prescribing and to discontinue unnecessary or unclear PPI therapy.

PPI deprescribing failure can be attributed to several factors. First, indications for short-term treatment were frequently adopted as long-standing “secondary diagnoses” in medical reports without proper reassessment of the need for continued PPI treatment. In discharge summaries, re-evaluation of the need for continued PPI treatment frequently was deferred to another healthcare professional post-discharge (e.g. general practitioner), without specifying the intended duration of PPI treatment. As a result, time constraints, communication gaps between healthcare providers and delayed or missed follow-up visits might have contributed to inappropriate long-term PPI use. Additionally, PPI prescriptions for patients who did not meet the criteria for bleeding prophylaxis point to uncertainty regarding appropriate treatment indications and highlights the need for improved prescriber education. These findings align with a qualitative study among general practitioners^50^ and a Swiss observational study conducted in primary care,^42^ both of which identified similar reasons for missed opportunities for deprescribing. In addition to our observations, these studies reported that deprescribing is often deprioritized in favor of managing more urgent acute conditions. Further barriers included patients' perceptions of PPIs as beneficial and fears of symptom recurrence, as well as reluctance to discontinue PPIs prescribed by specialists (e.g. gastroenterologists).^42^, ^50^ Therefore, in-hospital educational efforts^51^ and the use of computer-based tools, such as arriba-PPI,^52^ in primary care may improve the prescribing practices and should engage both healthcare providers and patients to support safe and effective PPI deprescribing.

A key strength of our study is that it provides a current overview of prescribing and deprescribing practices in hospital setting. The appropriateness of PPI use was evaluated based on national and international guidelines and the list of appropriate indications and therapy durations developed for this study could be widely applicable. The electronic health records were manually reviewed, instead of relying only on diagnostic codes, as they may lack accuracy for clinical diagnoses and therapy duration, and are driven by reimbursement requirements. A limitation of the study is that the results cannot be generalized as they are derived from a single hospital in central Switzerland introducing potential outcome bias. Replicating this study in other Swiss hospitals would reduce confounding effects related to language, geographical region, culture and patient care traditions in different cantons and could provide a broader overview of deprescribing practices. Furthermore, the sample size was low, limiting the statistical comparisons. Concomitant medication was assessed on admission and discharge without accounting for potential changes during hospitalization, thus not enabling an evaluation of criteria for bleeding prophylaxis related to other medications during hospitalization. Automated extraction of medication data across all phases of hospitalization should be considered allowing differentiation between regularly administered and as-needed medications that will reduce the time required for data verification and improve overall data extraction quality. The lowest age threshold used for the risk of bleeding prophylaxis (≥60 years instead of 65 used in some studies and guidelines) might have caused an overestimation of the appropriate PPI use. A standardized guideline for appropriate PPI use should be developed aligned with existing national and international guidelines. The retrospective design and manual review of health records may have introduced potential bias. Last but not least, access was restricted to hospital documentation and changes in the medication list between hospitalizations in the period of 6 months prior to hospitalization could not be verified, limiting the assessment of PPI duration prior to hospitalization. A prospective longitudinal study would help overcome the limitations associated with the retrospective design, while also enabling more accurate documentation of changes in PPI prescriptions and the assessment of potential PPI related adverse drug reactions.

## 5 Conclusions

Nearly all patients continued PPI use during hospitalization and at discharge, despite lacking a clearly documented indication in more than half of the cases. This highlights the frequent overprescription and insufficient deprescribing practices, likely due to poor documentation, communication gaps between health providers and time constraints. Regular medication reviews by general practitioners, community pharmacists and hospital clinicians could improve deprescribing practices and ensure appropriate PPI use and improve patient awareness of the risks of long-term PPI therapy, thereby increasing the acceptability and effectiveness of deprescribing.

## CRediT authorship contribution statement

**Christina Kotoula:** Writing – original draft, Visualization, Methodology, Formal analysis, Data curation. **Leonie Daria Schreck:** Writing – review & editing, Data curation. **Arani Anandarathakrishnan:** Writing – review & editing, Data curation. **Carla Meyer-Massetti:** Writing – review & editing, Methodology. **Carole Elodie Aubert:** Writing – review & editing, Methodology. **Manuel Haschke:** Writing – review & editing, Methodology. **Evangelia Liakoni:** Writing – review & editing, Methodology, Conceptualization.

## Funding

This research did not receive any specific grant from funding agencies in the public, commercial, or not-for-profit sectors. CEA was supported by the 10.13039/501100001711Swiss National Science Foundation (SNSF Ambizione grant PZ00P2_201672).

## Declaration of competing interest

The authors declare no conflict of interest regarding the publication of this article.

## Appendix A Supplementary data

Supplementary material Image 1
