# Supplementary material for: Deprescribing of proton pump inhibitors in internal medicine in-patients: A retrospective observational study
Source: Explor Res Clin Soc Pharm. 2026 May 7;23:100796. doi: 10.1016/j.rcsop.2026.100796 (PMC13200115; doi:10.1016/j.rcsop.2026.100796)
Supplement: Supplementary file 1 — Supplementary material [file mmc1.pdf]

# Deprescribing of proton pump inhibitors in internal medicine in-patients: a retrospective observational study

Christina Kotoula[1,2], Leonie Daria Schreck[1], Arani Anandarathakrishnan[1], Carla Meyer-Massetti[1,3], Carole Elodie Aubert[3,4], Manuel Haschke[1], Evangelia Liakoni[1]

*[1]Clinical Pharmacology and Toxicology, Department of General Internal Medicine, Inselspital, Bern University Hospital, University of Bern, Bern, Switzerland; [2]Graduate School for Health Sciences, University of Bern, Switzerland; [3]Institute of Primary Health Care (BIHAM), University of Bern, Bern, Switzerland; [4]Department of General Internal Medicine, Inselspital, Bern University Hospital, University of Bern, Bern, Switzerland*

**Corresponding author**: Christina Kotoula, Clinical Pharmacology & Toxicology, Inselspital, Bern University Hospital, Bern, Switzerland, christina.kotoula@students.unibe.ch

**Supplementary material**

**Supplemental Table S1:** List of documented appropriate indications across hospital stay. (PPI cases in each hospitalization phase: admission N=320, during hospitalization N=443, at discharge N=376).

| Indication | Admission<br>n (%) | Hospitalisation<br>n (%) | Discharge<br>n (%) |
|---|---|---|---|
| Helicobacter pylori (H. pylori) eradication (triple-therapy)[a]/ gastritis type B | 6 (2) | 6 (1) | 7 (2) |
| • Erosive gastroesophageal reflux disease (GERD) grade A or B according to Los Angeles classification<br>• Reflux symptoms, acid regurgitation, heartburn/stomach burn | 48 (15) | 67 (15) | 65 (17) |
| Non-erosive gastroesophageal reflux disease (NERD) | 0 | 0 | 0 |
| • Duodenal/ gastric ulcer<br>• Ulcer bleeding after endoscopy | 18 (6) | 29 (6) | 25 (7) |
| • NSAID[b] associated erosion/ gastritis type C<br>• Gastritis (unspecified), erosive antral gastritis, congestive gastritis | 26 (8) | 38 (9) | 33 (9) |

| | | | |
|---|---|---|---|
| • Upper abdominal complaints<br>• Dyspepsia (including functional acid-related dyspepsia)<br>• Irritable stomach<br>• Gastropathy | 9 (3) | 7 (2) | 7 (2) |
| Stress ulcer prophylaxis during intensive care unit stay | 0 | 1 (<1) | 0 |
| Axial hiatus hernia | 36 (11) | 52 (12) | 50 (13) |
| Esophageal varices | 10 (3) | 14 (3) | 13 (3) |
| Pathological hypersecretion (idiopathic, Zollinger-Ellison syndrome) | 0 | 0 | 0 |
| Barrett's esophagus | 21 (6) | 24 (5) | 24 (6) |
| Eosinophilic esophagitis | 0 | 0 | 0 |
| Peptic esophageal stricture/ stenosis | 1 (<1) | 1 (<1) | 1 (<1) |
| GERD grade C or D according to Los Angeles classification | 6 (2) | 7 (2) | 8 (2) |
| Prophylaxis of progression of idiopathic pulmonary fibrosis | 0 | 0 | 0 |
| Prophylaxis after relapse/recurrence after discontinuation with<br>➢ GERD<br>➢ Dyspepsia | 1 (<1) | 1 (<1) | 1 (<1) |
| Prevention of recurrence of idiopathic duodenal/ gastric ulcer | 1 (<1) | 3 (1) | 1 (<1) |
| Previous gastrointestinal ulcer bleeding | 14 (4) | 18 (4) | 17 (4) |

| | | | |
|---|---|---|---|
| Bleeding prophylaxis from the following combinations:<br><br>➢ NSAID (incl. aspirin and coxibs[c]) **AND** one of the following:<br>▪ Age ≥60 years<br>▪ Second NSAID<br>▪ Anticoagulants[d]<br>▪ Corticosteroids[e]<br>➢ Antiplatelet drugs[f] **AND** one of the following:<br>▪ Aspirin (dual antiplatelet therapy)<br>▪ Anticoagulant<br>▪ Age ≥60 years<br>▪ Corticosteroids<br>▪ GERD symptoms<br>▪ Dyspepsia<br>▪ NSAID | 136 (42) | n.a.[g] | 131 (35) |

[a]Triple therapy: PPI+ clarithromycin+ second antibiotic (amoxicillin or metronidazole)
[b]NSAID (non-steroidal anti-inflammatory drugs): acetylsalicylic acid (aspirin), diclofenac, ibuprofen, flurbiprofen, dexibuprofen, naproxen, indometacin, ketorolac, piroxicam, acemetacin, dexketoprofen, mefenamic acid, nimesulide, tenoxicam, etodolac
[c]Coxibs (COX-2-inhibitors): etoricoxib, celecoxib
[d]Anticoagulants: direct oral anticoagulants (dabigatran, rivaroxaban, apixaban, edoxaban), vitamin K antagonists (acenocoumarol, phenprocoumon), heparin incl. unfractionated & low molecular weight (dalteparin, enoxaparin, nadroparin, fondaparinux)
[e]Corticosteroids: betamethasone, budesonide, dexamethasone, hydrocortisone, methylprednisolone, prednisolone, prednisone, triamcinolone
[f]Antiplatelet drugs: acetylsalicylic acid (Aspirin), clopidogrel, prasugrel, ticagrelor, cangrelor, eptifibatid, tirofiban
[g]Co-medications reviewed on admission and at discharge but not during hospitalization.

**Supplemental Table S2:** Number of patients with different trajectories regarding the PPI use (duration and indication) in the alluvial plot

| **Admission** | **Hospitalization** | **Discharge** | **n (%)** |
|---|---|---|---|
| PPI inappropriate | PPI inappropriate | PPI inappropriate | 108 (24) |
| PPI appropriate | PPI appropriate | PPI appropriate | 106 (23) |
| No PPI | PPI inappropriate | PPI inappropriate | 44 (10) |
| No PPI | PPI appropriate | PPI appropriate | 42 (9) |
| PPI appropriate | PPI appropriate | PPI inappropriate | 28 (6) |
| No PPI | PPI inappropriate | No PPI | 26 (6) |
| No PPI | PPI appropriate | No PPI | 16 (4) |
| PPI inappropriate | PPI inappropriate | No PPI | 13 (3) |

| | | | |
|---|---|---|---|
| PPI inappropriate | PPI appropriate | PPI appropriate | 12 (3) |
| PPI appropriate | PPI appropriate | No PPI | 11 (2) |
| No PPI | PPI appropriate | PPI inappropriate | 10 (2) |
| PPI appropriate | No PPI | No PPI | 6 (1) |
| PPI inappropriate | No PPI | No PPI | 6 (1) |
| PPI inappropriate | PPI appropriate | PPI inappropriate | 6 (1) |
| PPI inappropriate | No PPI | PPI inappropriate | 3 (1) |
| PPI appropriate | PPI inappropriate | No PPI | 1 (<1) |
| PPI appropriate | PPI inappropriate | PPI inappropriate | 1 (<1) |
